# Applicability of Anatomic and Physiologic Scoring Systems for the Prediction of Outcome in Polytraumatized Patients with Blunt Aortic Injuries

**DOI:** 10.3390/diagnostics11112156

**Published:** 2021-11-21

**Authors:** Alexander Omar, Marcel Winkelmann, Emmanouil Liodakis, Jan-Dierk Clausen, Tilman Graulich, Mohamed Omar, Christian Krettek, Christian Macke

**Affiliations:** 1 Trauma Department, Hannover Medical School, 30625 Hannover, Germany; alexander.omar@me.com (A.O.); Winkelmann.Marcel@mh-hannover.de (M.W.); liodakis.emmanouil@mh-hannover.de (E.L.); Clausen.Jan-Dierk@mh-hannover.de (J.-D.C.); graulich.tilman@mh-hannover.de (T.G.); omar.mohamed@mh-hannover.de (M.O.); krettek.christian@mh-hannover.de (C.K.); 2 Bundeswehr Joint Medical Service, 26384 Wilhelmshaven, Germany

**Keywords:** trauma, aortic injury, scoring systems, multiple injured

## Abstract

Background: Most patients with blunt aortic injuries, who arrive alive in a clinic, suffer from traumatic pseudoaneurysms. Due to modern treatments, the perioperative mortality has significantly decreased. Therefore, it is unclear how exact the prediction of commonly used scoring systems of the outcome is. Methods: We analyzed data on 65 polytraumatized patients with blunt aortic injuries. The following scores were calculated: injury severity score (ISS), new injury severity score (NISS), trauma and injury severity score (TRISS), revised trauma score coded (RTSc) and acute physiology and chronic health evaluation II (APACHE II). Subsequently, their predictive value was evaluated using Spearman´s and Kendall´s correlation analysis, logistic regression and receiver operating characteristics (ROC) curves. Results: A proportion of 83% of the patients suffered from a thoracic aortic rupture or rupture with concomitant aortic wall dissection (54/65). The overall mortality was 24.6% (16/65). The sensitivity and specificity were calculated as the area under the receiver operating curves (AUC): NISS 0.812, ISS 0.791, APACHE II 0.884, RTSc 0.679 and TRISS 0.761. Logistic regression showed a slightly higher specificity to anatomical scoring systems (ISS 0.959, NISS 0.980, TRISS 0.957, APACHE II 0.938). The sensitivity was highest in the APACHE II with 0.545. Sensitivity and specificity for the RTSc were not significant. Conclusion: The predictive abilities of all scoring systems were very limited. All scoring systems, except the RTSc, had a high specificity but a low sensitivity. In our study population, the RTSc was not applicable. The APACHE II was the most sensitive score for mortality. Anatomical scoring systems showed a positive correlation with the amount of transfused blood products.

## 1. Introduction

Traumatic injuries have a high socioeconomic relevance and are the leading cause of death before the age of 45. During 2000 and 2010, the percentage of trauma deaths rose faster than the population increase in the United States [1]. Every trauma patient suffers from an individual combination of injuries, which leads to a variety of physiological impairments and post-traumatic complications during the clinical course. For an optimal outcome, an early risk stratification is important for therapy planning or the decision of patient transfer to a higher-level trauma center [2].

Trauma scoring systems are applied in trauma severity measurement, clinical decision making, outcome prediction and clinical scientific analysis. Scoring systems enable the classification and comparison of clinical courses and outcomes of trauma patients.

For trauma patients, triage scores can quantify the physiological impact of injuries’ severity, but they are not able to quantify the actual anatomic severity of injuries [3]. Therefore, trauma scoring systems should be universally applicable to different kinds of injury constellations. The variables used in scoring systems should be easy to measure and reliable. The predictive performance of the scoring systems is generally evaluated in a trauma patient population with a mix of different injury patterns. However, traumatic lesions of the great vessels are rare injuries, often related to high-energy trauma and therefore associated with multiply injured patients [4]. A problem with commonly used scoring systems could be that they are validated for hospitalized patients, whereas traumatic lesions of the aorta are associated with a high preclinical mortality [5,6]. A meta-analysis by Schimrigk et al. analyzed 14 publications with 136 patients who underwent an emergency thoracotomy. In 26% (*n* = 35/136) of the patients, a lesion of the great thoracic vessels was accountable for a traumatic cardiac arrest [7]. Severe injuries can have an impact on anatomic scoring systems, but are neglected by physiological scoring systems as long as they do not cause physiological impairment [8]. In patients with blunt aortic injuries, anatomic scoring systems may overestimate the influence on the outcomes of those patients. In contrast, physiological scoring systems take account of post-traumatic physiological impairment [9]. Trauma deaths show a trimodal distribution. Exsanguination and central nervous system injuries are the most frequent causes of death in acute and early trauma deaths, while organ failure is the predominant cause of late trauma deaths [10]. In the early period after trauma, an injury’s severity is the most important parameter regarding the outcome; later, the physiological impairment gains more relevance. Because of the high preclinical mortality, patients admitted alive to the emergency room are a selected group. The aim of our study was to evaluate and compare the performance of commonly used trauma scoring systems in mortality prediction for multiply injured patients with concomitant aortic injuries. Furthermore, we investigated the correlations of these scores with clinical outcome parameters (according to materials and methods 2.6) to evaluate the scores’ predictive performance.

## 2. Materials and Methods

### 2.1. Data Collection

**Figure 1:**
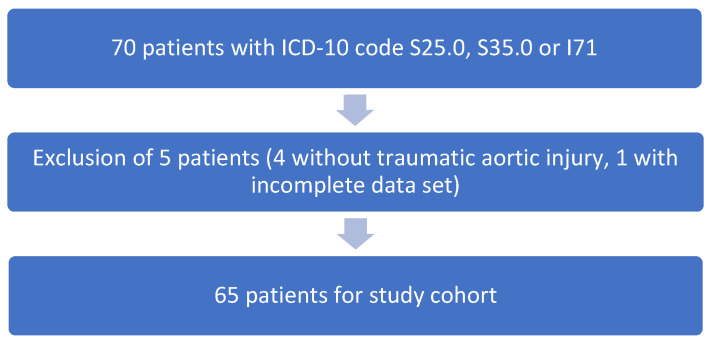
Recruitment flow diagram. We found 70 patients within our hospital databank. Four had to be excluded due to miss-coding and one excluded due to incomplete data.

For our study, we used the Hannover Medical School trauma databank. Patients in this study were selected by the International Classification of Diseases (ICD)−10 code S25.0 (Injury of thoracic aorta), S35.0 (Injury of abdominal aorta) and I71 (Aortic aneurysm and dissection). After the first selection, the patient data was screened using inclusion and exclusion criteria. A total of 65 patients were enrolled in our study (Figure 1).

### 2.2. Inclusion/Exclusion Criteria

We included polytrauma patients, defined by an injury severity score (ISS) ≥ 16, primarily or secondarily admitted within 72 h after trauma from 2003 to 2019 with complete data for retrospective analysis at our level−1 trauma center. 

### 2.3. Ethical Approval

The study followed the guidelines of the revised United Nations declaration of Helsinki in 1975 and its latest amendment in 2013. The study was approved by the Ethical Committee of Hannover Medical School (approval number 9118_BO_K_2020).

### 2.4. Scoring Systems

The degree of the physiological impairment and injury severity was measured by using the following scoring systems.

Anatomic scores:

- Abbreviated Injury Scale (AIS);
- Injury Severity Score (ISS);
- New Injury Severity Score (NISS).

Physiologic scores:

- Revised Trauma Score coded (RTSc);
- Acute Physiology and Chronic Health Evaluation II (APACHE II);
- Combined scores;
- Trauma and Injury Severity Score (TRISS).

The injury severity was classified according to the ISS and NISS, based on the AIS, which was introduced by the Association for the Advancement of Automotive Medicine [11,12,13].

For the classification of physiological impairment after trauma, the RTS and RTSc were used. These scores measure the neurological impairment with the Glasgow Coma Scale (GCS), circulatory impairment with the systolic blood pressure (SBP) and respiratory impairment with the respiratory rate (RR). The coding of the RTS values was done according to the formula: RTSc = 0.9368 × GCSc + 0.7326 × SBPc + 0.2908 × RRc [14]. Based on the RTSc and ISS, the trauma injury severity score (TRISS) was calculated [15]. 

Additionally, for the estimation of mortality the APACHE II score was used [16]. 

### 2.5. Score Calculation

For the calculation of the RTSc, initial vital parameters (the respiratory rate, systolic blood pressure and GCS) were used to avoid a bias from prehospital treatment.

Estimated survival by TRISS: $\frac{1}{1+1^{-\left(0.4499+0.8085*RTSc+\left(-0.0835\right)*ISS+\left(-1.743\right)\right)}}$

The following coefficients were used for calculating the survival:

- Blunt trauma coefficient, −0.4499;
- RTSc coefficient, 0.8085;
- ISS coefficient, −0.0835;
- Age coefficient (if age > 54 years): −1.743.

### 2.6. Clinical Course/Clinical Parameters

The total in-patient time (days), duration of intensive care unit (ICU) treatment (days), hours of ventilation time (VT), as well as the amount of the required transfusion of packed red blood cells (PRBC), fresh frozen plasma (FFP) and platelet concentrate (PC) in the first 48 h after trauma and during the total in-patient time, were recorded. 

### 2.7. Diagnosis and Management of Blunt Thoracic Aortic Injuries (BTAI)

After the admission of patients under stable conditions, initial trauma diagnostics including X-rays of the chest, pelvis, lateral spine and extremities (whenever necessary), as well as a computed tomography scan of the head, spine, chest, abdomen and pelvis, were carried out. For the diagnosis of vascular injuries, contrast enhanced CT-scans were performed and evaluated by a consultant radiologist. Patients in extremis received an emergency thoracotomy immediately, which revealed a thoracic aortic rupture. Aortic injuries were regularly diagnosed and classified with computed tomography according to the criteria defined by Dyer et al.: poorly defined fat planes, a mediastinal hemorrhage, perivascular hemorrhage, periaortic hematoma, change in the caliber of the aorta, intraluminal irregularities and an abnormal contour of the aorta or the proximal great vessels [17]. Patient management was led by the responsible trauma surgeon, in conjunction with a surgeon in the Department of Cardiothoracic, Transplantation and Vascular Surgery in the case of aortic injuries. The type of treatment of the aortic injury was dependent on the discretion of the responsible cardiothoracic/vascular surgeon.

### 2.8. Statistics

A statistical analysis was performed using the JASP (Version 0.13.1, JASP Team, Amsterdam, The Netherlands) and Statistical Package for the Social Sciences (SPSS) 27.0 produced by the International Business Machines Corporation (IBM, Chicago, IL, USA). A correlation analysis was performed by using Kendall’s tau-b and Spearman’s rho. Trauma score performance was analyzed by logistic regression. Based on the regression curves, the sensitivity, specificity and area under the curve (AUC) were calculated. Statistical significance was set at *p* ≤ 0.05.

## 3. Results

### 3.1. Demographic Data

In total, 65 multiple trauma patients with aortic injuries who met our inclusion parameters were enrolled in the study. Table 1 shows the parameters of male and female patients.

### 3.2. Mechanism of Injury

The most frequent injury mechanism in our study population was having an accident involving motorized vehicles (vehicle accidents 35/65 (53.9%), motorcycle accidents 15/65 (23.1%)). A frequent mechanism was a car hitting a tree or a frontal collision. Other trauma mechanisms were falling from great height 7/65 (10.8%), pedestrian/cyclist accidents 6/65 (9.2%) and traumatic body compression 2/65 (3.1%).

### 3.3. Aortic Injuries, Treatment of Blunt Aortic Injuries and Consequences of Stenting

Out of 65 patients with aortic injuries, 56 (86.2%) patients suffered from thoracic aortic ruptures or thoracic aortic ruptures with concomitant aortic dissections (Table 2). Only three patients showed an isolated aortic rupture without further damage of the aortic wall. Six patients had only minimal aortic injuries like wall hematomas or small intima flaps. 

### 3.4. Causes of Death

During the study, the overall mortality was 24.6% (16/65). The highest number of fatalities was observed during the early clinical phase, with 50% (8/16 patients) of the casualties occurring within the first 24 h (Table 3). Only four deaths were a direct consequence of the aortic injury. From day three, single and multiple organ dysfunction became an increasing cause of death. Multiple organ dysfunction was defined as an altered organ function of two or more organs, e.g., kidney failure with acidosis and pulmonary dysfunction. 

### 3.5. Logistic Regression

Logistic regressions were performed to evaluate the predictive performance of the different scoring systems regarding mortality (Table 4). All scores except the TRISS and RTSc showed significant results. Regarding performance, the anatomic, physiologic and combined scores had a high specificity and a low sensitivity. 

Considering the Pseudo-R^2^, the McFadden R^2^ values were as follows: NISS, 0.267; ISS, 0.256; ISS without aorta, 0.077; TRISS, 0.108; APACHE II, 0.354; and RTSc, 0.028. These results indicate a better fit in the logistic regression models of the APACHE II, NISS and ISS. The RTSc showed only a weak fit, which led to an unpredictable sensitivity and specificity.

### 3.6. Correlation Analysis of Scores

A correlation analysis was performed to evaluate the correlation between the NISS, ISS, APACHE II, TRISS, RTSc, GCS, shock index and clinical outcome parameters. The specific parameters were the total in-patient time, ventilation time and amount of transfused blood products. The rank correlation was measured using Kendall’s tau and Spearman’s rho (exact values are shown in the heatmaps in the Supplementary Materials). Kendall’s tau demonstrated a significant positive correlation between the NISS and ISS with the amount of transfused blood products. Furthermore, the NISS had a significant, but weak, negative correlation with the total in-patient time. The APACHE II score was used to calculate the estimated mortality, which showed a weak positive correlation with the total number of transfused platelet concentrates (PC). The TRISS-estimated survival negatively correlated with the transfused amount of blood products. The shock index showed only a weak correlation with the total number of PC and number of packed red blood cells (PRBC) in the first 48 h. 

Spearman’s rho revealed similar results. The only differences were found in the correlation of the TRISS-estimated survival with the amount of transfused blood products. 

Additionally, we calculated an ISS without considering aortic injuries. This modified ISS had a weak correlation with the ventilation time and a positive correlation with the amount of transfused blood products. The GCS did not correlate with hospitalization and the ventilation time. 

### 3.7. Receiver Operating Characteristics Curves

The predictive performance represented by the sensitivity and specificity of mortality was evaluated by comparisons of the area under the ROC curves. The TRISS results in a survival probability, and if a low RTS is associated with a higher mortality, then the ROC curves should lie close to zero. For better comparability, the ROC curves for the TRISS and RTS were inverted. 

**Figure 2:**
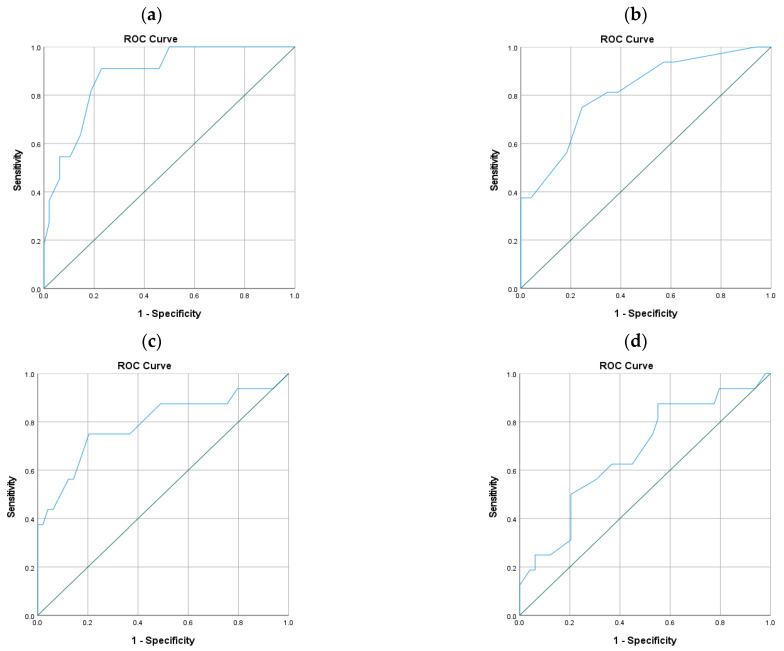

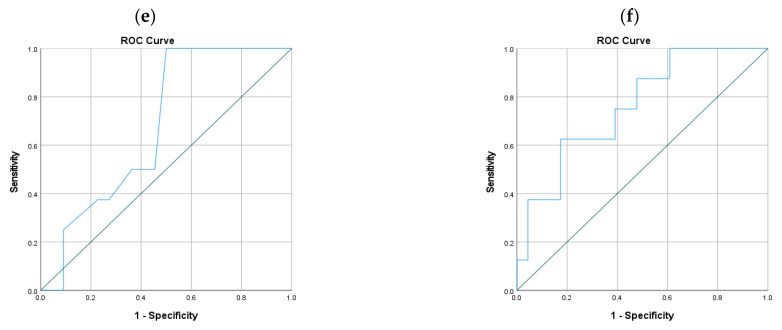
ROC curves for the (**a**) APACHE II, (**b**) NISS, (**c**) ISS, (**d**) ISS without aortic injuries, (**e**) RTSc and (**f**) TRISS. APACHE II—acute physiology and chronic health evaluation II; NISS—new injury severity score; ISS—injury severity score; RTSc—revised trauma score coded; TRISS—trauma and injury severity score.

Area under the ROC curve comparison (Figure 2):

- NISS, 0.812, (95%-CI, 0.689–0.935; asymptotic significance, 0.000*);
- ISS, 0.791 (95%-CI, 0.643–0.940; asymptotic significance, 0.001*);
- ISS without aortic injuries, 0.671 (95%-CI, 0.516–0.826; asymptotic significance 0.041*);
- APACHE II, 0.884 (95%-CI, 0.786–0.981; asymptotic significance 0.000*);
- RTSc, 0.679 (95%-CI, 0.486–0.872; asymptotic significance 0.140);
- TRISS, 0.761 (95%-CI, 0.577–0.945; asymptotic significance 0.030*);
- Shock index, 0.702 (95%-CI, 0.522–0.881; asymptotic significance 0.050).
  * significant

## 4. Discussion

### 4.1. Mortality

In the present study, commonly used trauma-scoring methods were compared for their performance in scoring polytraumatized patients with concomitant blunt aortic injuries. It is unclear whether aortic injuries can impair the prognostic qualities of the tested anatomic scoring systems. On the one hand, aortic injuries are highly graded in AIS-based scoring systems, but on the other hand, only 4 of 16 (25%) deaths in our study were caused directly by an aortic injury. Nevertheless, most deaths were injury-related and not the consequence of posttraumatic complications such as multiple organ dysfunction. 

Blunt traumatic injuries of the thoracic aorta are associated with a high preclinical mortality of approximately 85% and additional 30% during the first hours after admission [18,19]. This high early mortality correlates with the observations in our study of 50% (8/16 patients) of the deaths occurring on admission day. Most deaths occurred within the first three days. The cause of death in these patients was regularly a direct consequence of the sustained injuries. Only two patients died in a later period, at day 37 and 61. 

A high early mortality is typical in high-energy trauma, which was the most frequent injury mechanism in this study population. Evans et al. investigated 103 high-energy trauma deaths, 66% during the prehospital phase and 27% within the first 48 h after admission. The most common causes of death during this phase were exsanguination (33%) and central nervous system injuries (33%) [20]. 

### 4.2. Abbreviated Injury Scale-Based Anatomic Scoring Systems

Aortic injuries, even intimal tears, are graded ≥4 in AIS 2005, which results in high ISS and NISS in these patients [21]. The relevance of an aortic injury to a patient´s outcome ranges from intimal tears without active bleeding to acute life-threatening massive hemorrhage. Since many patients die in the prehospital phase, and only four patients in our study died in-hospital due to an aortic injury, such a high AIS grading is questionable as a prognostic factor. Furthermore, AIS-based scores have other disadvantages. A study by Aharonson-Daniel et al. demonstrated that an identical ISS/NISS, generated by different triplets, is associated with a different risk of mortality and that triplets including higher AIS values are associated with a higher inpatient mortality [22]. 

Secondly, in some injuries the AIS is coded by complications such as blood loss >20% of blood volume in pelvic ring fractures. In these cases, a differentiation between injury severity and injury mismanagement by AIS/ISS is impossible [23]. Despite these disadvantages, the ISS and NISS have some clinical relevance. Parimi et al. demonstrated that patients with a higher ISS will more likely need massive blood transfusion [24]. This result was also reproduceable in the selected population in our study. The ISS and NISS are directly correlated with the amount of transfused blood products. The exclusion of aortic injuries did not improve the predictive quality of AIS-based scores. The ROC and logistic regression showed a decreasing prognostic prediction for mortality when the aortic injury was excluded. 

Harwood et al. have compared AIS-based scoring systems and published areas under ROC curves of 0.785 (NISS) and 0.780 (ISS) for mortality prediction in blunt trauma patients [25]. These results are close to the results in our study with an area under the curve of 0.812 for NISS and 0.791 for ISS. 

The better predictive performance of the NISS can be explained by the inclusion of concomitant injuries in the same AIS-region, especially in a population with predominantly blunt injury mechanisms. Esmer et al. published a study with data on 30,777 patients from the German TraumaRegister DGU^®^ between 1993 and 2009, and found a median of 6.6 injuries per polytrauma patient, with 95% sustaining blunt trauma and 58% sustaining thoracic trauma [26]. Our study population confirmed these results, as an aortic injury was regularly associated with further thoracic injuries such as lung contusions (42/65 patients) and rib fractures (42/65 patients). Only two patients had no AIS-relevant additional thoracic injuries.

### 4.3. Physiologic Scoring Systems

The RTS comes in two versions—the T-RTS for triage and the RTSc for outcome evaluation. The RTSc coefficients place a high weight on the GCS and lower weights on the systolic blood pressure and respiratory rate [14]. The high weight on the GCS is comprehensible, because central nervous system injuries are a leading cause of death (21.6–71.5%) in multiply injured patients [27]. In our patient group, traumatic brain injuries caused 25% of the fatalities. Nonetheless, the RTS showed an underperformance in our study, and there could be various reasons for this. When calculating the RTSc we used the initial measured parameters, because these parameters were not influenced by prehospital therapy. Physiological parameters are more difficult to record, especially in a prehospital setting. Using the initial data, such as the respiratory rate, causes more missing data. In our study population, just 30 of 65 patients had complete data sets for RTSc calculation. The respiratory rate was the most frequent cause of a failed RTSc calculation. Only 32 of 65 patients had a preclinically documented respiratory rate, which was lower compared to the initial oxygen saturation, which was available for 43 of 65 patients. In the case of the 16 deaths in our study, only 8 patients supplied data for RTSc calculation, resulting in a non-significant sensitivity and specificity in the logistic regression and a low area under the ROC curve with broad confidence intervals. Therefore, the RTSc showed a relevant underperformance compared to the other trauma scoring systems. 

In trauma patients, the initial surgical therapy depends on the level of physiological impairment and complexity of the surgical procedures [28]. Therefore, an ideal scoring system should deliver results as early as possible. This is the major disadvantage of the APACHE II score; because of the observation period and variety of measured parameters, only 11/16 fatalities in our study had an APACHE II score. However, the APACHE II score demonstrated the highest sensitivity for mortality and the highest AUC of all tested scores (0.880). This AUC was close to the value published by Wu et al. of 0.892 [29]. Opposing our results, other studies found a possible superiority of the ISS, NISS and TRISS over the APACHE II for geriatric polytraumatized patients [30]. This demonstrates that the predictive quality of a scoring system depends on the underlying patient population. 

### 4.4. Combined Scoring Systems

The TRISS is trying to close the gap between anatomical and physiological scoring systems by adding the RTSc and age of the patient to the ISS. This should take into account physiological impairments, which are associated with the injuries and lower resistance of older patients. The literature reports a lower predictive performance of the TRISS compared to the APACHE II (Wu et al. 0.881 vs. 0.892; Agarwal et al. 0.813 vs. 0.885) [29,31], which was also confirmed by our results. The reason for this performance difference could be the underperformance of the RTSc, which impairs the TRISS [32]. The AUC of the TRISS in our study was notably lower (0.761), which was a result of the RTSc. The problem of missing RTS values for the TRISS was reported by Gabbe et al. in a review article (3–28% missing RTS) [32]. In most studies, which compare the predictive performances of different scores, patients with incomplete data for a score calculation were excluded. This would produce a selection bias. 

### 4.5. Limitations and Strength

An important limitation is the lack of data for the RTS calculation in our study population. This could be a cause for the underperformance of the RTS/ TRISS, but otherwise the elimination of those patients could cause a selection bias. For a further evaluation of the predictive performance of the RTS and TRISS, a prospective multicenter study would be beneficial to avoid a bias caused by missing data. A prospective study would take time, due to the incidence of 0.7% blunt traumatic aortic injuries in all trauma patients [33]. 

The major strength of this study is that, to the best of our knowledge, it is the largest single-center study investigating the applicability of these scoring systems for patients with blunt traumatic aortic injuries.

## 5. Conclusions

The results of the presented study demonstrate the applicability of the ISS, NISS, APACHE II and TRISS to multiply injured patients with concomitant aortic injuries, but the predictive abilities of all scoring systems were very limited. In summary, we suggest the ISS for the initial assessment and the addition of the APACHE II during the initial intensive care treatment. A major advantage of anatomic scoring systems was their correlation with the amount of transfused blood products. Even though the aortic injury was not associated with a relevant bias in AIS-based scores, the APACHE II was a better predictor of mortality. 

## Figures and Tables

**Table 1:** Demographic data of the enrolled patients.

Parameter	Study Population	
	Male	Female
Number (*n*)	56	9
Age (years); mean ± SD	41.4 (±17.5)	43.3 (±20.5)
Sex; *n* (%)	56 (86.2%)	9 (13.8%)
ISS; median (IQR)	34 (18.25)	45 (17)
NISS; median (IQR)	41 (17.75)	50 (16)
RTS; median (IQR)	6.904 (1.874)	3.867 (0.733)
TRISS; median (IQR)	82.75 (60.27)	7.64 (11.67)
APACHE II; mean median (IQR)	22 (11.5)	22 (9)
ICU time (days); median (IQR)	13 (19)	24 (24)
Hospitalization time (days); median (IQR)	19.5 (19.75)	24 (24)
Ventilation (hours); median (IQR)	230 (442)	397 (555)
Mortality; *n* (%)	12 (21.4%)	4 (44.4%)

NISS—new injury severity score; ISS—injury severity score; TRISS—trauma and injury severity score; APACHE II—acute physiology and chronic health evaluation II; RTS—revised trauma score; ICU—intensive care unit; SD—standard deviation; IQR—interquartile range.

**Table 2:** Type and frequency of the aortic injuries as well as the performed treatment.

Types of Aortic Injuries	Frequency *n* (%)	Open Surgery *n* (%)	Endovascular Surgery *n* (%)	Conservative Therapy *n* (%)	Mortality *n* (%)
Aortic wall hematoma	4 (6.2)	0 (0)	0 (0)	4 (100)	0 (0)
Aortic wall rupture	38 (58.5)	6 (15.8)	27 (71.1)	5 (13.2)	11 (29.0)
Aortic dissection	3 (4.6)	0 (0)	2 (66.7)	1 (33.3)	0 (0)
Comb. rupture and dissection	16 (24.6)	3 (18.8)	12 (75.0)	1 (6.3)	4 (25.0)
Intimaflap	2 (3.1)	0 (0)	0 (0)	2 (100)	0 (0)
Abdominal aortic injury	1 (1.5)	1 (100)	0 (0)	0 (0)	0 (0)
Thoracic plaque rupture	1 (1.5)	0 (0)	0 (0)	1 (100)	1 (100) *
Overall	65 (100)	10 (15.39)	41 (63.08)	14 (21.54)	16 (24.62)

* A therapy limitation due to the patient’s advance directive.

**Table 3:** Causes of death for all patients in this study.

Number	Sex	Death after (days)	Cause of Death	ISS
1	female	1	hemorrhagic shock due to retroperitoneal bleeding	45
2	male	1	secondary free aortic rupture	75 ^a,b^
3	female	1	secondary free aortic rupture	75 ^a,b^
4	male	1	traumatic brain injury	75
5	male	1	secondary free aortic rupture	75 ^a,b^
6	male	1	secondary free aortic rupture	75 ^a,b^
7	male	1	traumatic brain injury	45
8	male	1	hemorrhage after left pulmonary hilus rupture	75 ^a^
9	male	2	myocardial infarction caused by coronary artery disease	26
10	male	2	Traumatic brain injury	45
11	male	2	hypotension (therapy limitation due to advance healthcare directive)	21
12	male	3	respiratory failure	50
13	male	3	multiple organ dysfunction syndrome	34
14	female	8	traumatic brain injury	57
15	male	37	multiple organ dysfunction syndrome	50
16	female	61	multiple organ dysfunction syndrome	36

ISS—injury severity score; ^a^ at least one AIS of 6; ^b^ death as direct consequence of aortic injury.

**Table 4:** Comparison of all trauma scores in view of the sensitivity, specificity and predictive performance of the different scoring systems regarding mortality.

Score	AUC	Sensitivity	Specificity	Odds Ratio	95% Confidence Interval (ORS)
NISS	0.812	0.375	0.980	1.117	0.050–0.171 *
ISS	0.791	0.438	0.959	1.096	0.041–0.142 *
ISS w/o aorta	0.671	0.125	1.000	1.059	0.007–0.107 *
TRISS	0.761	0.250	0.957	0.977	−0.047–0.001
APACHE II	0.880	0.545	0.938	1.089	0.034–0.136 *
RTSc	0.679	0.000	1.000	0,802	–0.623–0.221

AUC—area under the curve; ORS—odds ratio scale; NISS—new injury severity score; ISS—injury severity score; TRISS—trauma and injury severity score; APACHE II—acute physiology and chronic health evaluation II; RTSc—revised trauma score coded; * significant.

## Data Availability

The data presented in this study are available on request from the corresponding author. The data are not publicly available, because they were collected from the clinic´s database.

## Supplementary Materials

The following are available online at https://www.mdpi.com/article/10.3390/diagnostics11112156/s1, Heatmap 1 Kendall’s tau: Correlation between Scores and clinical outcome parameters. Heatmap 2 Spearman’s rho: Correlation between Scores and clinical outcome parameters.
