# Supplementary material for: Applicability of Anatomic and Physiologic Scoring Systems for the Prediction of Outcome in Polytraumatized Patients with Blunt Aortic Injuries
Source: Diagnostics (Basel). 2021 Nov 21;11(11):2156. doi: 10.3390/diagnostics11112156 (PMC8617692; doi:10.3390/diagnostics11112156)
Supplement: Supplementary file 1 [file diagnostics-11-02156-s001.zip › diagnostics-1405157-supplementary.pdf]

Heatmap 1 Kendall's tau: Correlation between Scores and clinical outcome parameters.

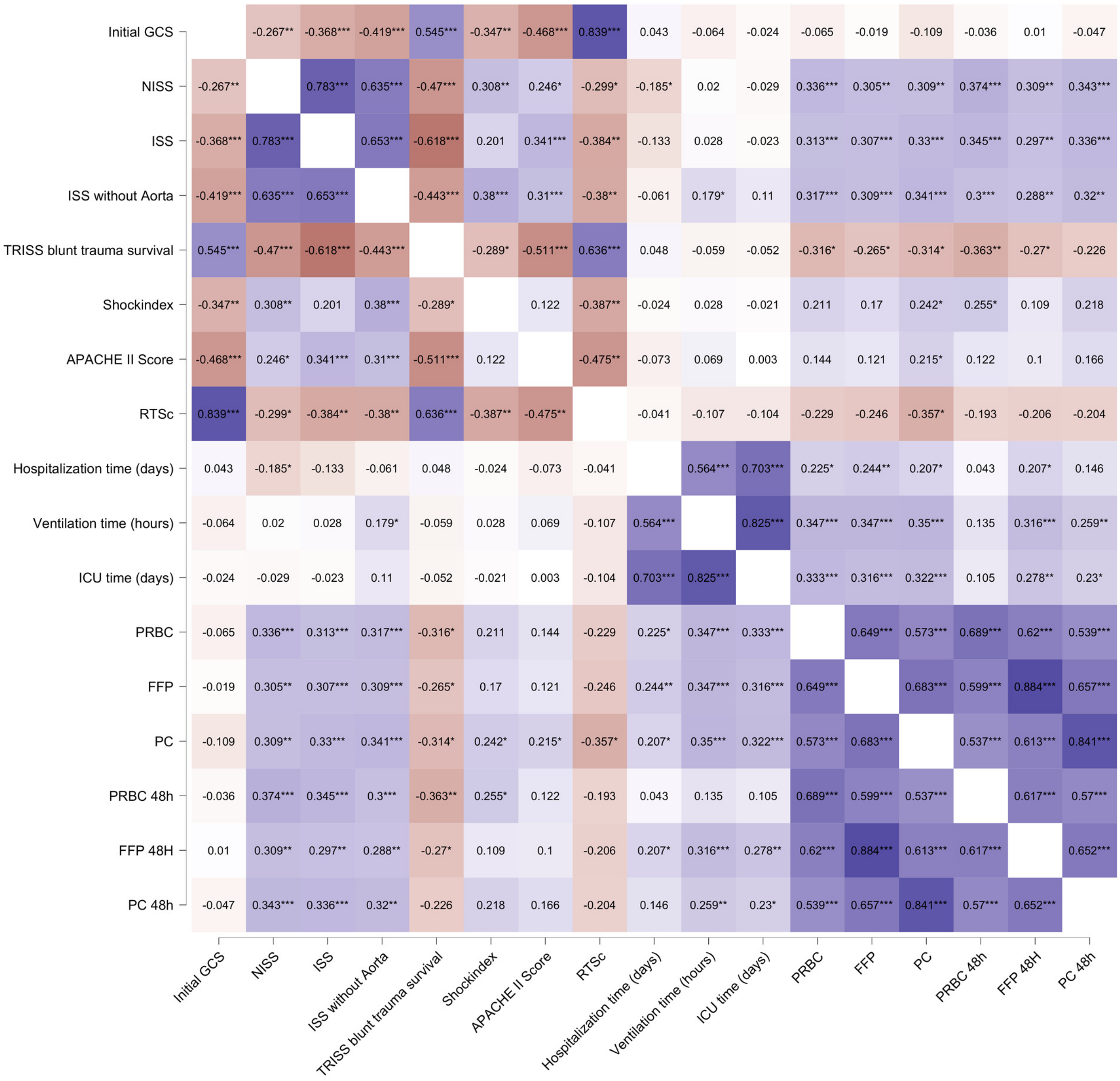

GCS= Glasgow Coma Scale; NISS: New Injury Severity Score, ISS: Injury Severity Score; TRISS: Trauma and Injury Severity Score; APACHE II: Acute Physiology And Chronic Health Evaluation II; RTSc: Revised Trauma Score coded; ICU time: Intensive Care Unit time; PRBC: Packed Red Blood Cells; FFP: Fresh Frozen Plasma; PC: platelet concentrates; *p < .05, **p < .01, ***p < .001

Heatmap 2 Spearman's rho: Correlation between Scores and clinical outcome parameters.

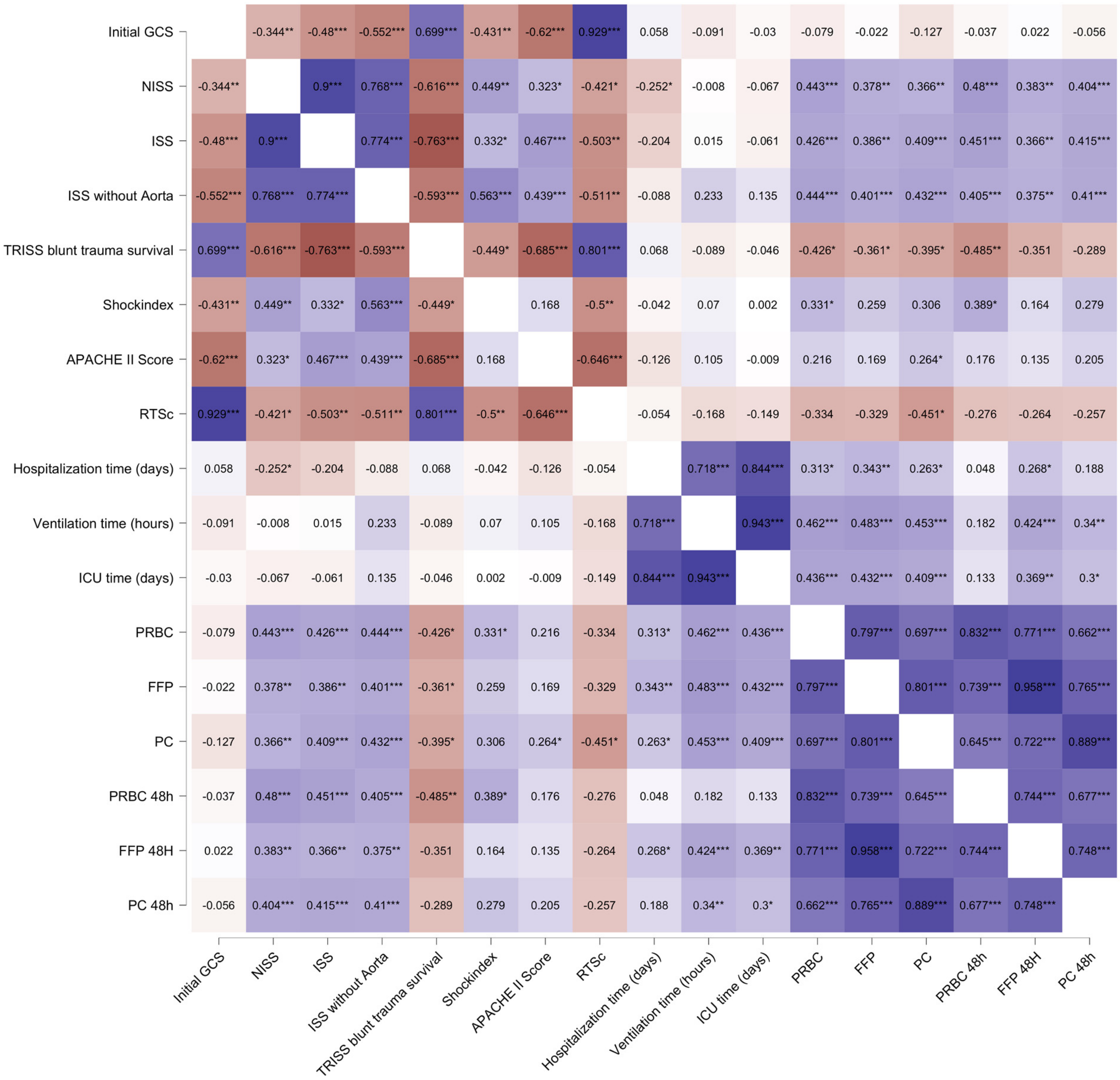

GCS= Glasgow Coma Scale; NISS: New Injury Severity Score, ISS: Injury Severity Score; TRISS: Trauma and Injury Severity Score; APACHE II: Acute Physiology And Chronic Health Evaluation II; RTSc: Revised Trauma Score coded; ICU time: Intensive Care Unit time; PRBC: Packed Red Blood Cells; FFP: Fresh Frozen Plasma; PC: platelet concentrates; $*p < .05$, $**p < .01$, $***p < .001$.
